# Trans-Species Polymorphism in Mitochondrial Genome of Camarodont Sea Urchins

**DOI:** 10.3390/genes10080592

**Published:** 2019-08-05

**Authors:** Evgeniy S. Balakirev

**Affiliations:** 1 National Scientific Center of Marine Biology, Far Eastern Branch, Russian Academy of Sciences, 17 Palchevsky Street, 690041 Vladivostok, Russia; esbalakirev@mail.ru; Tel.: +7-423-231-0905 or +7-423-243-3280; 2 School of Biomedicine, Far Eastern Federal University, 8 Sukhanov Street, 690950 Vladivostok, Russia

**Keywords:** sea urchins, Camarodonta, mitochondrial genome, *ND6* gene, trans-species polymorphism, transcriptome, short read archive database, Eocene/Oligocene boundary, global ocean temperature change

## Abstract

Mitochondrial (mt) genomes of the sea urchins *Strongylocentrotus intermedius* and *Mesocentrotus nudus* demonstrate the identical patterns of intraspecific length variability of the *ND6* gene, consisting of 489 bp (S variant) and 498 bp (L variant), respectively. For both species, the *ND6* length difference is due to the 488A>G substitution, which changes the stop codon TAG in S variant for a tryptophan codon TGG in L variant and elongates the corresponding ND6 protein by three additional amino acids, Trp-Leu-Trp. The phylogenetic analysis based on mt genomes of sea urchins and related echinoderm groups from GenBank has shown the S and L *ND6* variants as shared among the camarodont sea urchins; the rest of the echinoderms demonstrate the S variant only. The data suggest that the *ND6* 488A>G substitution can be the first example of the trans-species polymorphism in sea urchins, persisting at least since the time of the Odontophora diversification at the Eocene/Oligocene boundary (approximately 34 million years ago), which was characterized by an abrupt climate change and significant global ocean cooling. Alternative hypotheses, including the convergent RNA editing and/or codon reassignment, are not supported by direct comparisons of the *ND6* gene sequences with the corresponding transcripts using the basic local alignment search tool (BLAST) of full sea urchin transcriptomes.

## 1. Introduction

*Strongylocentrotus intermedius* (A. Agassiz, 1863) and *Mesocentrotus nudus* (A. Agassiz, 1863) are sea urchin species common in the Northwest Pacific [1,2]. These species are distributed mostly from the littoral and upper sublittoral zones to a depth of 25 m [3]. The species represent two major clades (*Strongylocentrotus* and *Mesocentrotus*) of the strongylocentrotid sea urchins (order Camarodonta, family Strongylocentrotidae) identified by genetic studies [4,5,6]. The estimated time of divergence between the *Strongylocentrotus* and *Mesocentrotus* clades is around 8.5–19 million years ago (MYA) [6,7,8]. Interspecies crosses between *S. intermedius* and *M. nudus* have been successfully performed in a laboratory [9,10], which is not unusual for sea urchins (review in [11]).

Little is known about the population genetics of *S. intermedius* and *M. nudus*; the available data are limited mostly to allozyme [12,13,14,15], mitochondrial, and microsatellite [16] markers. Using allozyme markers, Zaslavskaya et al. [15] studied the population genetic structure of *S. intermedius* in the northwestern Sea of Japan. The study revealed a significant genetic heterogeneity of sea urchin settlements without clear geographical pattern. The authors suggested that the genetic heterogeneity may be presumably a result of both genetic drift and natural selection [15]. Using microsatellite and mtDNA markers, Nam et al. [17] studied 10 natural settlements of *M. nudus* around the Japanese islands and the Korean Peninsula. They did not find any genetic heterogeneity and assumed the recent population expansion (about 5000 to 10,000 years ago) to be a cause of the lack of genetic divergence between the present settlements [17].

Previously, we detected two significantly diverged (*F*_ST_ = 0.671; *p* < 0.01) haplotype groups in the *COI* gene (lineage 1 and lineage 2) of *S. intermedius* collected from three geographical localities of the Sea of Japan [18,19]. We have recently expanded the analysis and sequenced two complete *S. intermedius* mt genomes (GenBank accession numbers KY964299.1 and KY964300.1) [15] and detected a gene arrangement similar to that of other sea urchin mt genomes published previously (see Tale S1 for references and GenBank accession numbers). The two *S. intermedius* mt genomes were equal in length (15,705 bp) and similar in nucleotide sequences (*D*_xy_ = 0.0083 ± 0.0007) with the only length difference found within the *ND6* gene. A conceptual translation revealed equal amino acid lengths of 12 mt proteins except ND6, which consisted of 162 (short, S variant) and 165 (long, L variant) amino acids in the two genomes studied. This length difference was due to the 488A>G substitution (numbered from the beginning of the *ND6* gene), which changed the termination codon TAG in S variant for a tryptophan codon TGG in L variant and elongated the corresponding ND6 protein by three additional amino acids, Trp-Leu-Trp. The increased sample of the *ND6* sequences (GenBank accession numbers KY964290.1–KY964298.1) revealed five S and four L variants, showing a high level of the 488A>G polymorphism in *S. intermedius*. Consequently, there may be two variants of the ND6 protein. The two putative variants share the amino acid residues 1–162 but have different C-terminal ends depending on the removal or not of a three-amino acid tail, Trp-Leu-Trp.

In the current study, the complete mt genomes and separate *ND6* gene sequences of sea urchins and other echinoderm groups, obtained from GenBank, were analyzed to clarify the ND6 L/S polymorphism detected in *S. intermedius*. The *ND6* DNA sequences were compared with the corresponding RNA transcripts using the Short Read Archive (SRA) sequence database of the National Center for Biotechnology Information (NCBI) to distinguish between the alternative hypotheses explaining the *ND6* intra- and interspecific patterns of variability among sea urchins.

## 2. Materials and Methods

The complete mt genomes and separate *ND6* sequences of sea urchins (Echinoidea), sea stars (Asteroidea), brittle stars (Ophiuroidea), sea cucumbers (Holothuroidea), and sea lilies (Crinoidea) were downloaded from the GenBank NCBI (https://www.ncbi.nlm.nih.gov/) [20] genetic sequence database (Flat File Release 231.0, 15 April 2019) (see Table S1 for accession numbers). The nucleotide and protein sequences were aligned using the MUSCLE [21] and MAFFT v. 7 [22] software. The DnaSP v. 5 [23] and PROSEQ v. 2.9 [24] programs were used for intra-and interspecific analyses. Phylogeny reconstructions were based on the full mt genome sequence alignment using the neighbor-joining and maximum-likelihood methods in MEGA v. 7 [25]. MEGA7 or jModelTest [26] were used to find the best-fit model of nucleotide substitution under the maximum likelihood criterion. The general time reversible + gamma + invariant sites (GTR + G + I) model showed the lowest Bayesian information criterion (BIC; [27]) score (407,026.784) and Akaike information criterion (AIC; [28]) value (406,267.663) and was chosen for further phylogenetic reconstructions. Maximum likelihood bootstrap analyses [29] consisted of 500 replicates. 

The complete dataset was also analyzed by Bayesian inference using MrBayes v. 3.2.7a (released 6 March 2019; [30]) under the GTR + G + I model with the default run length (1,000,000 generations). The proportion of invariable sites was uniformly distributed over the interval (0.00, 1.00). Gamma distribution was approximated using four categories. Analyses were performed as two independent runs, each with four incrementally heated metropolis-coupled Monte Carlo–Markov chains. Output trees and data were sampled for every 500 generations. Likelihood values reached a plateau within 15,000 generations. A total of 4002 trees in two files were read and 3002 of them were sampled. The number of unique site patterns was 8571. The log likelihood values increased from below −360,656.289 to about −234,790.217 in the first 5000 generations and then to about −234,634.892 after one million generations. The likelihood of the best state for “cold” chain of run 1 was −234,617.92 and the likelihood of the best state for a “cold” chain of run 2 was −234,618.40. The average standard deviation of split frequencies was 0.000269 after 1,000,000 generations indicating stationary conditions. A convergence diagnostic, the potential scale reduction factor (PSRF) [31] was between 1.000 and 1.006 for all parameters, thus, indicating a good sample from the posterior probability distribution.

The tree topologies for the camarodont sea urchins obtained by the neighbor-joining and maximum-likelihood methods, as well as by the Bayesian inference, were very similar. The close congruence could be explained by the facts that the dataset was relatively straightforward and included only complete mt genome sequences of sea urchins. The quality of alignment was high, and the length of alignment was long enough (a total of 19.1 kb). As has been shown by many authors, the relative efficiencies of different methods applied for obtaining the correct tree topology are very close under these conditions (e.g., [32,33]). The difference between the methods applied to the camarodont sea urchins (our dataset) resulted in slightly different topologies and bootstrap values and had no changes concerning the distribution of the *ND6* L and S variants. To be conservative, we provide the lowest bootstrap values obtained by the maximum-likelihood method.

Physicochemical features of the putative S and L variants of the ND6 protein were computed using the Protein Feature Server (PROFEAT) [34]. Full sea urchin transcriptomes were searched with the BLAST algorithm [35] using the NCBI Short Read Archive sequence database (https://www.ncbi.nlm.nih.gov/sra/) [36].

## 3. Results and Discussion

**Figure 1:**
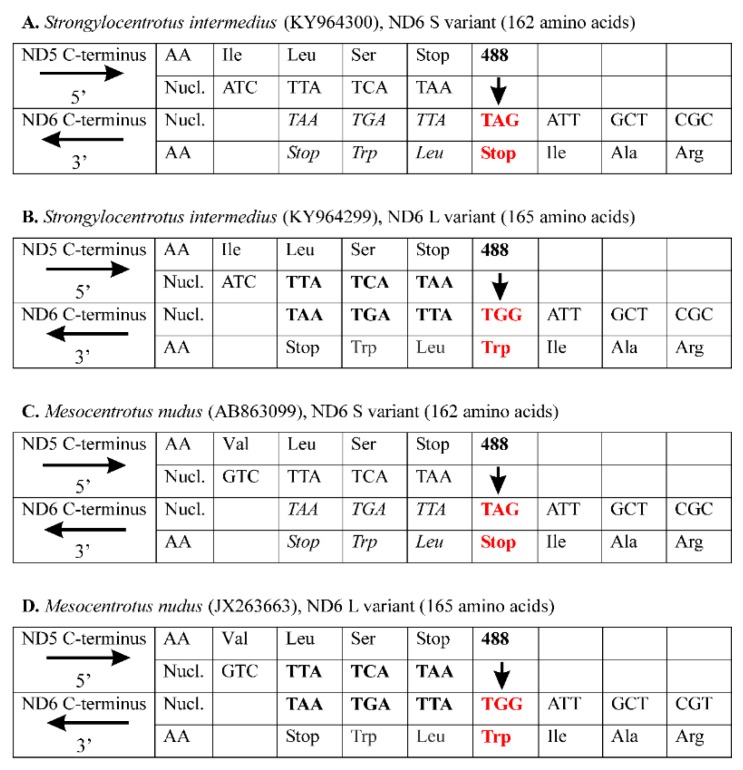
The ND5 and ND6 C-terminus sequences of the sea urchins *Strongylocentrotus intermedius* (**A**,**B**) and *Mesocentrotus nudus* (**C**,**D**). Conceptual translation of the *ND5* and *ND6* genes is shown as three-letter abbreviations (echinoderm mitochondrial genetic code). The *ND5* gene is encoded on the H-strand, and the *ND6* gene is encoded on the L-strand. The strands and corresponding transcripts are denoted by horizontal arrows → (H strand) and ← (L strand). The 5’ and 3’ ends are shown on the left. For the L variant, there is an overlap (highlighted in bold) between ND5 and ND6 encoded on the opposite strand. Nucl. is nucleotides; AA is amino acid residues. The *ND6* polymorphic site 488A>G is indicated by bold vertical arrows under the numeral 488; the corresponding codons (TAG and TGG) are highlighted in bold red type indicating the changes in S and L variants. The readthrough extension for the S variants is highlighted in italics.

Figure 1 shows the *S. intermedius* and *M. nudus* C-terminal amino acid and the corresponding nucleotide sequences for the adjacent *ND5* and *ND6* genes, which are encoded on the opposite DNA strands with the opposite transcript orientation. To illustrate the pattern of the ND6 variability, we arbitrarily present the last three for S variant and six for L variant of C-terminal amino acids along with a termination codon of the *ND6* gene, as well as the last three C-terminal amino acids along with a termination codon of the *ND5* gene. For *S. intermedius*, there is no overlap between the *ND5* and *ND6* genes for the S variant (Figure 1A), however there is a 9-bp overlap for the L variant (Figure 1B). The length difference is due to the 488A>G substitution (indicated by vertical bold arrow), which changes the *ND6* stop codon TAG in S variant for a tryptophan codon TGG in L variant (highlighted in red bold type), adding a 9-bp downstream elongation TTATGATAA (including a termination codon TAA). The conceptually translated proteins consist of 162 and 165 amino acids for the S and L variants, respectively. The L variant is by three amino acids longer than the S variant due to the C-terminal three-amino acid tail, Trp-Leu-Trp. Consequently, there may be two variants of the ND6 protein. The two putative variants share the amino acid residues 1–162 but have different C-termini depending on the removed or not three-amino acid tail, Trp-Leu-Trp (Figure 1A,B).

The same pattern of *ND6* intraspecific variability has been found in the other sea urchin, *Mesocentrotus nudus*, whose multiple *ND6* sequences are available in GenBank (accession numbers AB863097.1–AB863112.1; [37]). For this species, there are two main *ND6* variants consisting of 162 and 165 amino acids, and the length difference results from the same polymorphic site (488A>G), which determines a termination codon TAG in S variant (Figure 1C) but a tryptophan codon TGG and subsequent downstream elongation TTATGATAA in L variant (Figure 1D). The nucleotide elongation encodes an identical Trp-Leu-Trp C-terminal tail of the ND6 L variant for both sea urchin species (Figure 1). Thus, *S. intermedius* and *M. nudus* demonstrate the identical patterns of intraspecific *ND6* L/S length polymorphism. Five S and four L variants have been recorded from *S. intermedius* (GenBank accession numbers KY964290.1–KY964298.1); two S and 14 L variants, from *M. nudus* (GenBank accession numbers AB863097.1–AB863112.1; [37]). Another length variant of the *ND6* sequence has been detected in *M. nudus* (AB863103.1), which is longer than other sequences belonging to the L variant due to two additional amino acids, Asn–Asp [37].

**Figure 2:**
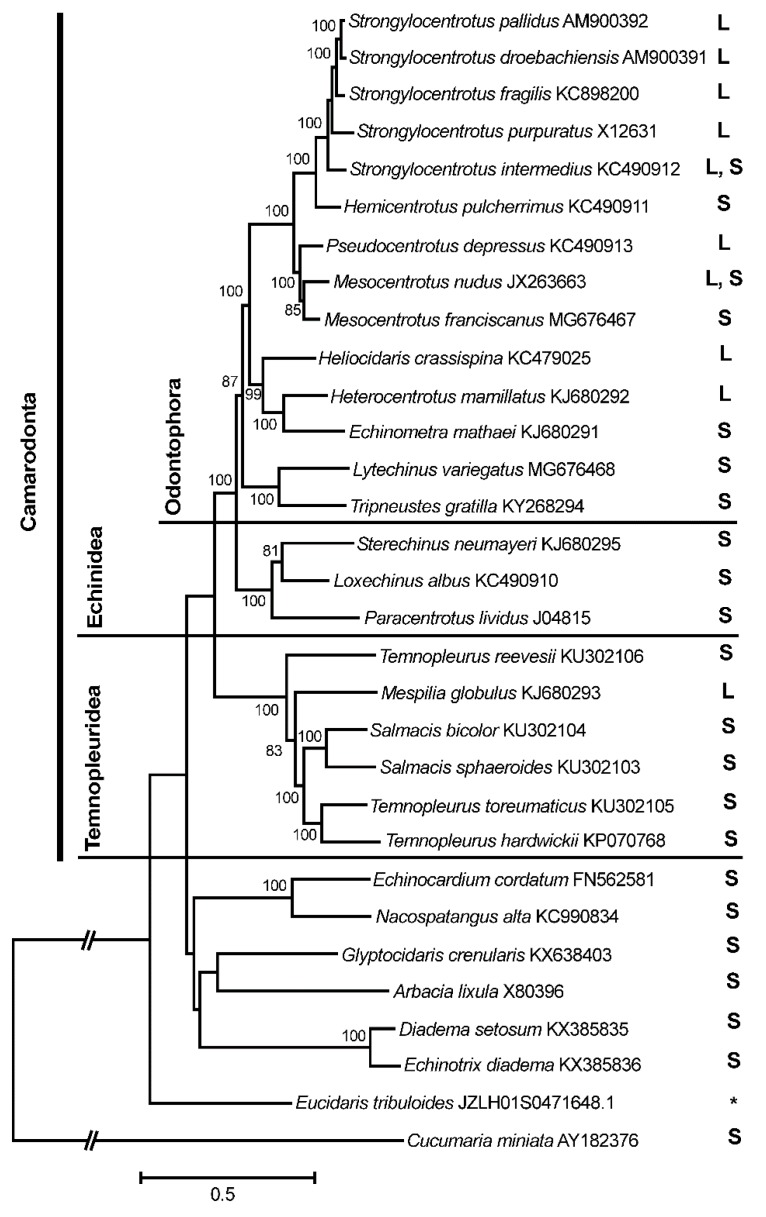
A maximum likelihood tree constructed using complete mitochondrial genomes of sea urchins (class Echinoidea). The tree is based on the general time reversible + gamma + invariant sites (GTR + G + I) model of nucleotide substitution. The mitochondrial genome of *Cucumaria miniata* (AY182376; class Holothuroidea) is used as an outgroup. The numbers at the nodes are bootstrap percentage probability values based on 500 replications (values below 75% are omitted). The species above the first (on top) horizontal line belong to the superfamily Odonthophora; above the second horizontal line, to the infraorder Echinidea; above the third horizontal line, to the order Camarodonta including the infraorders Echinidea and Temnopleuridea. The *ND6* L and S variants are indicated by the corresponding letters on the right: L, long (165 amino acids) *ND6* variant; S, short (162 amino acids) *ND6* variant; L, S, both *ND6* variants detected for *Strongylocentrotus intermedius* and *Mesocentrotus nudus*. The *Eucidaris tribuloides ND6* variant (indicated by asterisk) is shorter than the S variant of other sea urchins due to the deletion of seven amino acids at the C-terminus (see Figure S1).

To clarify the phylogenetic distribution of the observed *ND6* variability, the complete mt genomes and separate *ND6* gene sequences of sea urchins and other echinoderm groups available in GenBank were analyzed. Figure 2 shows a maximum likelihood phylogenetic tree based on the complete mt genomes of 30 sea urchin species. The topology of the tree is in congruence with the recent most comprehensive morphological and phylogenetic analyses (e.g., [38,39,40,41,42,43,44] and references therein). The ND6 L and S variants (indicated by the corresponding letters, Figure 2, right) are shared among the sea urchins of the order Camarodonta, including mostly the members of the superfamily Odontophora [8,38,43]. Outside Odontophora, the L variant is found in *Mespilia globulus* (infraorder Temnopleuridea) only (Figure 2). The members of the sea urchin orders Spatangoida (*Echinocardium cordatum* and *Nacospatangus alta*), Stomopneustoida (*Glyptocidaris crenularis*), Arbacioida (*Arbacia lixula*), and Diadematoida (*Diadema setosum* and *Echinotrix diadema*) (Figure 2), as well as other echinoderm classes including Asteroidea, Ophiuroidea, Holothuroidea, and Crinoidea (see Table S1 for species names and accession numbers), demonstrate the *ND6* S variant only, suggesting its ancestral state. The *ND6* gene of the sea urchin *Eucidaris tribuloides* (order Cidaroida) is truncated in such a way that the corresponding ND6 protein lacks seven amino acids of the S-terminus (Figure S1); the species was excluded from further analysis.

The *ND6* terminus is highly conserved in all the sea urchins studied (Figure S1, Table S2). The elongation of the *ND6* L variant includes the same three amino acid residues, Trp-Leu-Trp, in most species except *Heterocentrotus mammillatus* and *Mespilia globulus* having Leu–Leu–Trp and Leu–Leu–Cys, respectively (Table S2). The high functional constraint of the *ND6* C-terminal region could be explained by the binding site for the transcription factor mtDBP located at the junction between the *ND5* and *ND6* genes [45,46]. The transcription factor regulates the interplay between mtDNA transcription and replication in mitochondria. The binding sites of the mtDBP are conserved between the sea urchins *Paracentrotus lividus* and *Strongylocentrotus purpuratus* (review in [46]). The regulatory network architecture of the developmental genes is highly conserved in sea urchins (e.g., [41,42,47,48]).

Thus, the data have revealed a shared intra- and interspecific variability at the *ND6* gene among the camarodont sea urchins. It is unlikely that the *ND6* length polymorphism can be explained by a sequencing problem associated with sea urchins, because identical results were obtained by different authors investigating different sea urchin species (see Table S1). The pattern can represent an example of trans-species polymorphism (TSP), persisting at least since the time of the Odontophora sea urchin diversification at the Eocene/Oligocene boundary (approximately 34 MYA) [8,38,43]. TSP refers to the occurrence of identical or similar alleles maintained by the balancing selection in related species, except for the instances where similarity arose through convergence or introgression [49,50,51]. TSP arises when multiple allelic lineages that have originated in an ancestral species are maintained in descendant species. Assuming that multiple ancestral allelic variants are adaptive, their polymorphism, shared by descendant species, may be maintained by balancing selection for longer periods of time beyond neutral expectations [52]. TSP was frequently reported for the immune genes (the major histocompatibility complex, MHC), where balancing selection can maintain ancestral allelic variants for millions of years after the divergence (review in [53,54,55]). Until present, no TSP phenomenon has been reported for sea urchins.

The ND6 protein is crucial for the assembly and function of complex I (NADH: quinone oxidoreductase) of the mitochondrial electron transport chain that produces approximately 40% of the proton-motive force required to synthesize adenosine triphosphate essential for vital functions reviews in [56,57,58,59]. Respiratory complex I performs the reduction of quinone to quinol and pumps protons across the inner membrane of mitochondria. The ND6 protein is an indispensable part of the quinone binding site and even a single or a few amino acid mutations in ND6 may affect quinone binding and dynamics causing severe pathologies [60,61,62,63,64,65,66,67,68]. The residue substitutions that occur in this complex are assumed to interfere with the efficiency of the proton-pumping process, which then influences metabolic performance.

In spite of the strong functional constraint, the complex I genes have been considered important in the adaptive evolution of mitochondrial genome (e.g., [69,70]). The ND6 protein evolution was found to be under positive selection in cephalopod molluscs [71], Alvinocaridid shrimps [72], mosquitoes [73], grasshoppers [74], cichlid fishes [75], Antarctic nototheniid fishes [76], toad-headed lizards [77], songbirds [78], alpine pheasants [79], African elephants [80], Chinese domestic and Tibetan horses [81,82,83], and Chinese snub-nosed monkeys [84]. A significant departure from neutrality in the *ND6* gene was also reported for humans [85]. The data suggest that the *ND6* modifications may improve protein conformation and, therefore, the entire complex I subunit interactions. Consequently, the ND6 amino acid length difference, detected in the present study, can be an important source for adaptive variation in sea urchins.

The two putative ND6 protein variants, L and S, have the same amino acid sequences, except for the Trp-Leu-Trp C-terminus tail found in the L variant. The tail contains two tryptophan residues, which could be a radical change because the ND6 protein is relatively short in sea urchins (162–165 amino acids), and, except the Trp-Leu-Trp tail, there is only a single tryptophan residue in the ND6 protein alignment (position 95, Figure S1). Indeed, the two additional tryptophan residues lead to a significant difference in amino acid composition between the two putative ND6 variants (Wilcoxon test, *p* = 0.0181), which, in turn, explain the marginally significant difference in hydrophobicity (Wilcoxon test, *p* = 0.0580) and the highly significant differences in physicochemical features estimated by the Grantham [86] chemical distance (Wilcoxon test, *p* < 0.001) and the Schneider and Wrede [87] physicochemical distance (Wilcoxon test, *p* < 0.001). 

As an alternative, we can suggest that the *ND6* gene length difference could however not be translated to the protein level if mt transcripts undergo to convergent RNA editing independently in different sea urchin lineages. The term “RNA editing” is defined as targeted modifications to RNAs that result in sequence differences, via nucleotide insertions/deletions or substitutions, between the transcriptome and the corresponding genomic sequences [88]. RNA editing is found in evolutionarily distant groups including viruses, bacteria, protists, fungi, plants, and animals including humans (e.g., review in [89,90,91,92]). It is a mechanism generating an adaptive protein sequence diversity and, thus, providing organisms with the opportunity to express diverse, functionally distinct, protein isoforms (e.g., [93,94]). Despite RNA editing has not yet been described from echinoderms’ mitochondria, it is suggested as a potential source of sequence diversification of the nuclear *Transformer* gene family involved in the immune function of the sea urchins *S. purpuratus* [95,96,97] and *Heliocidaris erythrogramma* [98].

As another alternative hypothesis, we can suggest that the TAG codon (Figure 1A,C) has been reassigned as an amino acid-encoding codon in sea urchins and does not signal translation termination anymore. This is not without precedent, as the stop-to-leucine and stop-to-alanine TAG reassignments were previously reported from mt genomes of several green algae of the order Sphaeropleales [99,100] and the chytrid fungus *Spizellomyces punctatus* and its relatives [101]. As in the case of the “RNA editing” alternative hypothesis (see above), the reassignment of a stop codon would result in translation of the 3′-UTR, which can produce potentially toxic extended proteins [102,103,104].

Finally, the shared phylogenetic distribution of the L and S *ND6* variants might reflect historical hybridization event(s) between sea urchins, which might have resulted in interspecific recombination of their mt DNA (as it has been found for many other organisms; e.g., [105,106] and references therein). Therefore, we analyzed the ND6 alignments for evidence of recombination using various recombination detection methods implemented in the program RDP4 [107]. All methods failed to reveal any signal of recombination between the *ND6* sequences (*p* > 0.05), thus rejecting hybridization as a possible explanation of the shared *ND6* polymorphism among sea urchins.

Recently, large arrays of RNA sequence data have been generated and stored in public repositories such as the Short Read Archive sequence database (www.ncbi.nlm.nih.gov/sra/). Many full sea urchin mt genomes (see Table S1) and transcriptomes are there available [108,109,110,111,112,113,114,115,116]. To assess directly the alternative hypotheses outlined above, we compared the *ND6* genomic sequences with the corresponding *ND6* transcripts using the BLAST algorithm [35]. In case a RNA-DNA difference is found, it would indicate a possible role for the RNA editing and/or codon reassignment in generating the shared ND6 S/L variability; otherwise, the alternative hypotheses cannot be accepted.

We performed a BLAST search of full transcriptomes (for accession numbers see Table S1) for all sea urchin species except *Eucidaris tribuloides* (see above). The BLAST search revealed the *ND6* RNA sequences to completely match that of DNA, which excluded convergent RNA editing and/or codon reassignment as alternative hypotheses, explaining the shared ND6 polymorphism among sea urchins. Moreover, both S and L *ND6* RNA transcripts were detected in *S. intermedius* and *M. nudus* (Figure S2), which agrees with the intraspecific *ND6* gene variability reported for these species (see above). Thus, the alternative hypotheses to explain the shared intra- and interspecific *ND6* polymorphism among sea urchins, including convergent RNA editing and/or codon reassignment, are not supported by direct comparisons of the *ND6* DNA sequences with the corresponding *ND6* RNA transcripts.

The data suggest that the *ND6* 488A>G substitution can be the first example of TSP in sea urchins, persisting since the time of the Odontophora diversification at the Eocene/Oligocene boundary (approximately 34 MYA; [8,38,43]). The Eocene/Oligocene boundary was characterized by a significant global ocean cooling [117] associated with the largest extinctions of marine invertebrates in the Cenozoic era [118,119]. At the same time, the major global climate changes caused an extensive floral and faunal turnover (e.g., [120]) and species diversification inferred, for instance, for sea urchins [8], ostracods [121], diatoms [122], and whales [123]. Thus, the two variants of the *ND6* gene probably represent a genetic adaptation to the abrupt climate change and significant global ocean cooling and were maintained by the long-lasting balancing selection driven by the climate transition at the Eocene/Oligocene boundary. It has been suggested that the thermal environment is likely to impose particularly strong selection on the mt DNA sequences [124,125], and there is experimental evidence that the thermal selection indeed shapes the mt genome evolution of the fruit fly *Drosophila melanogaster* [126], which is consistent with the “mitochondrial climatic adaptation” hypothesis (review in [127]).

The L *ND6* variant found in *Mespilia globulus* (infraorder Temnopleuridea; Figure 2, Figure S1, Table S2) suggests that the *ND6* L/S TSP could be even more ancient than the Odontophora diversification time because the infraorders Echinidea and Temnopleuridea might share a common ancestor more than 100 MYA [38]. The indispensable function of the *ND6* protein and the likely signature of positive selection in other organisms (see above) suggest potential functional consequences for the *ND6* S and L variants of sea urchins. Anyway, taking into account that the putative *ND6* L/S TSP has been maintained over at least 34 million years of echinoid evolution, it is likely that the *ND6* variants could have significant consequences for the sea urchin thermal adaptation and, therefore, they deserve further investigations. 

## Supplementary Materials

The following are available online at https://www.mdpi.com/2073-4425/10/8/592/s1: Figure S1. (DOC format: doc). Sea urchin ND6 protein alignment. Figure S2. (DOC format: doc). Short Read Archive sequence database search of the *Strongylocentrotus intermedius* and *Mesocentrotus nudus* transcriptomes. Table S1. (DOC format: doc). GenBank accession numbers. Table S2. (DOC format: doc). Sea urchin ND6 C-terminal sequences. DOI: 10.5281/zenodo.3352428.
