# Supplementary material for: Trans-Species Polymorphism in Mitochondrial Genome of Camarodont Sea Urchins
Source: Genes (Basel). 2019 Aug 5;10(8):592. doi: 10.3390/genes10080592 (PMC6723515; doi:10.3390/genes10080592)
Supplement: Supplementary file 1 [file genes-10-00592-s001.zip › Figure_S2.doc]

**A.** SRX5702317: *Strongylocentrotus intermedius* ND6 L variant

```
Sequence ID: SRA:SRR8920978.14870267.1

Query  24   TTTTTTAATC-TTA-TCA-TAA-CCA-AAT-AGC-GCG-GA  83
            ||||||||||-|||-|||-|||-|||-|||-|||-|||-||
Sbjct  1    TTTTTTAATC-TTA-TCA-TAA-CCA-AAT-AGC-GCG-GA  60
                        *   W   L   W   I   A   R
```

**B.** SRX5702315: *Strongylocentrotus intermedius* ND6 S variant

```
Sequence ID: SRA:SRR8920980.27057861.1

Query  145  TTTTAATC-TTA-TCA-TAA-CTA-AAT-AGC-GCG-G  174
            ||||||||-|||-|||-|||-|||-|||-|||-|||-|
Sbjct  30   TTTTAATC-TTA-TCA-TAA-CTA-AAT-AGC-GCG-G  1
                      *   W   L   *   I   A   R
```

**C.** SRX2345657: *Mesocentrotus nudus* ND6 L variant

```
Sequence ID: SRA:SRR5017174.28968832.2

Query  130  TTTTAGTC-TTA-TCA-TAA-CCA-AAT-AGC-ACG-AATTAT  189
            ||||||||-|||-|||-|||-|||-|||-|||-|||-||||||
Sbjct  65   TTTTAGTC-TTA-TCA-TAA-CCA-AAT-AGC-ACG-AATTAT  6
                      *   W   L   W   I   A   R
```

**D.** SRX3652395: *Mesocentrotus nudus* ND6 S variant

```
Sequence ID: SRA:SRR6675841.2893993.2

Query  17   TTA-TCA-TAA-CTA-AAT-AGC-ACG-AAT-TAT-TGT  44
            |||-|||-|||-|||-|||-|||-|||-|||-|||-|||
Sbjct  122  TTA-TCA-TAA-CTA-AAT-AGC-ACG-AAT-TAT-TGT  149
             *   W   L   *   I   A   R
```

**Figure S2. Short Read Archive sequence database full transcriptomes search.**

The full sea urchin transcriptomes were searched with the BLAST algorithm (Altschul et al. 1990) using the NCBI Short Read Archive sequence database (https://www.ncbi.nlm.nih.gov/sra/). The figure present the ND5 termination codons (first on the left, denoted by *) and the ND6 C-terminal end for the L variant (W–L–W–I–A–R) and the S variant (I–A–R) with the corresponding termination codons (*). A. *Strongylocentrotus intermedius* ND6 L variant (query KY964299). B. *Strongylocentrotus intermedius* ND6 S variant (query KY964300). C. *Mesocentrotus nudus* ND6 L variant (query JX263663.1). D. *Mesocentrotus nudus* ND6 S variant (query AB863099). Conceptual translation of the ND6 gene is shown in one-letter abbreviations (echinoderm mitochondrial genetic code). The ND5 gene is encoded on the H-strand and the ND6 gene is encoded on the L-strand. The sense sequence of the ND6 gene is on the L-strand; thus, the nucleotide sequence is derived from the complementary sequence of the H-strand shown. The terminal codon TAG in S variant and a tryptophan codon TGG in L variant are in bold face. The readthrough extension is highlighted in italics. See Figure 1 and Table S2 for additional explanations.

**References**

Altschul, S.; Gish, W.; Miller, W.; Myers, E.; Lipman, D. Basic local alignment search tool. *J. Mol. Biol.* 1990, *215*, 403–410.
