# Supplementary material for: Trans-Species Polymorphism in Mitochondrial Genome of Camarodont Sea Urchins
Source: Genes (Basel). 2019 Aug 5;10(8):592. doi: 10.3390/genes10080592 (PMC6723515; doi:10.3390/genes10080592)
Supplement: Supplementary file 1 [file genes-10-00592-s001.zip › Table_S1.DOC]

**Table S1.** The GenBank accession numbers for the echinoderms studied at the present work

**Class Echinoidea**

| Species | GenBank accession number | Reference | ND6 variant |
| --- | --- | --- | --- |
| *Arbacia lixula* | X80396.1 | De Giorgi et al. 1996 | S |
| *Diadema setosum* | KX385835.1 | Li et al. 2016 | S |
| *Echinocardium cordatum* | FN562581.1 | Perseke et al. 2010 | S |
| *Echinometra mathaei* | KJ680291.1 | Jung et al. 2017 | S |
| *Echinometra sp. EZ* | MH685644.1 | Ketchum et al. 2018 | S |
| *Echinothrix diadema* | KX385836.1 | Li et al. 2017 | S |
| *Eucidaris tribuloides* | JZLH01S0471648.1 | Murali et al. 2015 | * |
| *Glyptocidaris crenularis* | KX638403.1 | Ding et al. 2017 | S |
| *Heliocidaris crassispina* | KC479025.1 | Jung et al. 2016 | L |
| *Hemicentrotus pulcherrimus* | KC490911.1 | Jung et al. 2014 | S |
| *Hemicentrotus pulcherrimus* | KC898202.2 | Kober and Bernardi 2013 | S |
| *Heterocentrotus mamillatus* | KJ680292.1 | Jung et al. 2017 | L |
| *Loxechinus albus* | KC490910.1 | Jung and Lee 2015 | S |
| *Loxechinus albus* | JX888466.1 | Cea et al. 2015 | S |
| *Lytechinus variegatus* | MG676468.1 | Bronstein and Kroh 2018 | S |
| *Lytechinus variegatus* | MG676469.1 | Bronstein and Kroh 2018 | S |
| *Mesocentrotus franciscanus* | KC898199.2 | Kober and Bernardi 2013 | S |
| *Mesocentrotus franciscanus* | KJ526170.1 | Gaitan-Espitia and Hofmann 2016 | S |
| *Mesocentrotus franciscanus* | MG676467.1 | Bronstein and Kroh 2018 | S |
| *Mesocentrotus nudus* | JX263663.1 | Jung et al. 2013 | L |
| *Mesocentrotus nudus* | KC898201.2 | Kober and Bernardi 2013 | L |
| *Mesocentrotus nudus* | AB863097.1 | Nam et al. 2013 | L |
| *Mesocentrotus nudus* | AB863098.1 | Nam et al. 2013 | L |
| *Mesocentrotus nudus* | AB863099.1 | Nam et al. 2013 | S |
| *Mesocentrotus nudus* | AB863100.1 | Nam et al. 2013 | S |
| *Mesocentrotus nudus* | AB863101.1 | Nam et al. 2013 | L |
| *Mesocentrotus nudus* | AB863102.1 | Nam et al. 2013 | L |
| *Mesocentrotus nudus* | AB863103.1 | Nam et al. 2013 | L |
| *Mesocentrotus nudus* | AB863104.1 | Nam et al. 2013 | L |
| *Mesocentrotus nudus* | AB863105.1 | Nam et al. 2013 | L |
| *Mesocentrotus nudus* | AB863106.1 | Nam et al. 2013 | L |
| *Mesocentrotus nudus* | AB863107.1 | Nam et al. 2013 | L |
| *Mesocentrotus nudus* | AB863108.1 | Nam et al. 2013 | L |
| *Mesocentrotus nudus* | AB863109.1 | Nam et al. 2013 | L |
| *Mesocentrotus nudus* | AB863110.1 | Nam et al. 2013 | L |
| *Mesocentrotus nudus* | AB863111.1 | Nam et al. 2013 | L |
| *Mesocentrotus nudus* | AB863112.1 | Nam et al. 2013 | L |
| *Mespilia globules* | KJ680293.1 | Jung et al. 2017 | L |
| *Nacospatangus altus* | KC990834.1 | Park et al. 2013 | S |
| *Paracentrotus lividus* | J04815.1 | Cantatore et al. 1989 | S |
| *Pseudocentrotus depressus* | KC490913.1 | Jung and Lee 2013 | L |
| *Pseudocentrotus depressus* | KC898203.2 | Kober and Bernardi 2013 | L |
| *Salmacis bicolor rarispina* | KU302104.1 | Wu et al. 2017c | S |
| *Salmacis sphaeroides* | KU302103.1 | Wu et al. 2017a | S |
| *Sterechinus neumayeri* | KF214257.1 | Dilly et al. 2015 | S |
| *Sterechinus neumayeri* | KJ680295.1 | Jung, et al. 2017 | S |
| *Strongylocentrotus fragilis* | KC898200.2 | Kober and Bernardi 2013 | S |
| *Strongylocentrotus intermedius* | KC490912.1 | Jung and Lee 2013 | L |
| *Strongylocentrotus intermedius* | KC898198.2 | Kober and Bernardi 2013 | L |
| *Strongylocentrotus intermedius* | KY964299.1 | Balakirev et al. 2017 | L |
| *Strongylocentrotus intermedius* | KY964300.1 | Balakirev et al. 2017 | S |
| *Strongylocentrotus intermedius* | KY964290.1 | Balakirev et al. 2017 | S |
| *Strongylocentrotus intermedius* | KY964291.1 | Balakirev et al. 2017 | S |
| *Strongylocentrotus intermedius* | KY964292.1 | Balakirev et al. 2017 | L |
| *Strongylocentrotus intermedius* | KY964293.1 | Balakirev et al. 2017 | L |
| *Strongylocentrotus intermedius* | KY964294.1 | Balakirev et al. 2017 | S |
| *Strongylocentrotus intermedius* | KY964295.1 | Balakirev et al. 2017 | L |
| *Strongylocentrotus intermedius* | KY964296.1 | Balakirev et al. 2017 | S |
| *Strongylocentrotus intermedius* | KY964297.1 | Balakirev et al. 2017 | L |
| *Strongylocentrotus intermedius* | KY964298.1 | Balakirev et al. 2017 | S |
| *Strongylocentrotus droebachiensis* | AM900391.1 | Andersen and Moum 2016 | L |
| *Strongylocentrotus droebachiensis* | EU054306.1 | Rhee et al. 2012 | L |
| *Strongylocentrotus droebachiensis* | KC898196.2 | Kober and Bernardi 2013. | L |
| *Strongylocentrotus pallidus* | AM900392.1 | Andersen and Moum, 2016 | L |
| *Strongylocentrotus pallidus* | KC898197.2 | Kober and Bernardi 2013 | L |
| *Strongylocentrotus purpuratus* | X12631.1 | Jacobs et al. 1988 | L |
| *Temnopleurus hardwickii* | KP070768.1 | Fu et al. 2014 | S |
| *Temnopleurus reevesii* | KU302106.1 | Wu et al. 2017b | S |
| *Temnopleurus toreumaticus* | KU302105.1 | Wu et al. 2017c | S |
| *Tripneustes gratilla* | KJ680294.1 | Jung et al. 2017 | S |
| *Tripneustes gratilla* | KY268294.1 | Laruson 2017 | S |

**Class** Asteroidea

| Species | GenBank accession number | Reference | ND6 variant |
| --- | --- | --- | --- |
| *Echinaster brasiliensis* | MG636999.1 | Seixas et al. 2019 | S |
| *Acanthaster brevispinus*, | AB231476.1 | Yasuda et al. 2006 | S |
| *Acanthaster planci* | AB231475.1 | Yasuda et al. 2006 | S |
| *Freyastera benthophila* | MG563681.1 | Mu et al. 2018 | S |
| *Pisaster ochraceus* | X55514.2 | Smith 2003 | S |
| *Asterias amurensis* | AB183559.1 | Matsubara et al. 2005 | S |
| *Aphelasterias japonica* | KM244709.1 | Tang et al. 2014 | S |
| *Patiria pectinifera* | D16387.1 | Asakawa et al. 2016 | S |
| *Astropecten polyacanthus* | AB183560.1 | Matsubara et al. 2005 | S |
| *Luidia quinaria* | AB183558.1 | Matsubara et al. 2005 | S |
| *Echinaster brasiliensis* | MG636999.1 | Seixas et al. 2019 | S |

**Class** Holothuroidea

| Species | GenBank accession number | Reference | ND6 variant |
| --- | --- | --- | --- |
| *Apostichopus japonicus* | EU294194.1 | Shen et al. 2009 | S |
| *Holothuria scabra* | KP257577.1 | Xia et al. 2015 | S |
| *Holothuria forskali* | FN562582.1 | Perseke et al. 2010 | S |
| *Parapostichopus nigripunctatus* | AB525762.1 | Sasaki, Hamaguchi 2012 | S |
| *Apostichopus parvimensis* | KU168761.1 | Zhang et al. 2015 | S |
| *Apostichopus californicus* | KP398509.1 | Liu 2015 | S |
| *Stichopus horrens* | HQ000092.1 | Fan et al. 2011 | S |
| *Cucumaria miniata* | AY182376.1 | Scouras et al. 2004 | S |
| *Peniagone* sp. YYH-2013 | KF915304.1 | Huo et al. 2014 | S |
| *Scotoplanes* sp. TT-2017 | LC416624.1 | Tokano et al. 2018 | S |

**Class** Ophiuroidea

| Species | GenBank accession number | Reference | ND6 variant |
| --- | --- | --- | --- |
| *Ophiopholis aculeata* | AF314589.1 | Smith et al. 2000 | S |
| *Ophiocomina nigra* | FN562577.1 | Perseke et al. 2010 | S |
| *Astrospartus mediterraneus* | FN562580.1 | Perseke et al. 2010 | S |
| *Ophiura luetkenii* | AY184223.1 | Scouras et al. 2004 | S |
| *Amphipholis squamata* | FN562578.1 | Perseke et al. 2010 | S |
| *Ophiacantha linea* | KC990833.1 | Park et al. 2013 | S |
| *Ophiura albida* | AM404180.1 | Perseke et al. 2008 | S |

**Class Crinoidea**

| Species | GenBank accession number | Reference | ND6 variant |
| --- | --- | --- | --- |
| *Phanogenia gracilis* | DQ068952.1 | Scouras and Smith 2006 | S |
| *Neogymnocrinus richeri* | DQ068951.1 | Scouras and Smith 2006 | S |
| *Florometra serratissima* | AF049132.1 | Scouras and Smith 2001 | S |
| *Antedon mediterranea* | AM404181.1 | Perseke et al. 2008 | S |

**Sea urchin transcriptomes**

| Species | GenBank accession number | ND6 variant |
| --- | --- | --- |
| *Arbacia punctulata* | SRX1391905 | S |
| *Arbacia lixula* | SRX1470204 | S |
| *Diadema setosum* | SRX4383684 | S |
| *Echinocardium_cordatum* | SRX565112 | S |
| *Echinometra mathaei* | SRX4383680 | S |
| *Hemicentrotus pulcherrimus* | DRX100872 | S |
| *Loxechinus albus* | SRX4222063 | S |
| *Lytechinus variegatus* | SRX446166 | S |
| *Mesocentrotus franciscanus* | SRX5847744 | S |
| *Mesocentrotus franciscanus* | SRX847758 | S |
| *Mesocentrotus franciscanus* | SRX847760 | S |
| *Mesocentrotus franciscanus* | SRX847764 | S |
| *Mesocentrotus franciscanus* | SRX5653410 | S |
| *Mesocentrotus franciscanus* | SRX5653411 | S |
| *Mesocentrotus nudus* | SRX2345657 | L |
| *Mesocentrotus nudus* | SRX2345658 | L |
| *Mesocentrotus nudus* | SRX2345659 | L |
| *Mesocentrotus nudus* | SRX2345660 | L |
| *Mesocentrotus nudus* | SRX2347278 | L |
| *Mesocentrotus nudus* | SRX2347279 | L |
| *Mesocentrotus nudus* | SRX2347280 | L |
| *Mesocentrotus nudus* | SRX2347281 | L |
| *Mesocentrotus nudus* | SRX3529721 | L |
| *Mesocentrotus nudus* | SRX3529722 | L |
| *Mesocentrotus nudus* | SRX3529723 | L |
| *Mesocentrotus nudus* | SRX3529725 | L |
| *Mesocentrotus nudus* | SRX3529726 | L |
| *Mesocentrotus nudus* | SRX3652395 | S |
| *Paracentrotus lividus* | SRX766113 | S |
| *Sterechinus neumayeri* | SRX846970 | S |
| *Strongylocentrotus intermedius* | SRX5702317 | L |
| *Strongylocentrotus intermedius* | SRX5689921 | L |
| *Strongylocentrotus intermedius* | SRX5702315 | S |
| *Strongylocentrotus droebachiensis* | SRX1428004 | L |
| *Strongylocentrotus droebachiensis* | SRX1427970 | L |
| *Strongylocentrotus purpuratus* | SRX5493395 | L |
| *Strongylocentrotus purpuratus* | SRX5650452 | L |
| *Tripneustes gratilla* | SRX3800284 | S |

The *Eucidaris tribuloides* ND6 variant (indicated by *) is shorter that the S variant of other sea urchins due to seven amino acids deletion of the C-terminal end (see Fig. S1).

**References**

Andersen, A.; Moum, T. Mitochondrial DNA sequence evolution among closely related strongylocentrotid sea urchins. Unpublished.

Asakawa, S.; Himeno, H.; Miura, K.; Watanabe, K. Nucleotide sequence and gene organization of the starfish Asterna pectinifera mitochondrial genome. Unpublished.

Balakirev, E.S.; Pavlyuchkov, V.A.; Ayala, F.J. Complete mitochondrial genome of the phenotypically-diverse sea urchin *Strongylocentrotus intermedius* (Strongylocentrotidae, Echinoidea). *Mitochondrial DNA Part B: Res.* **2017**, *2*, 613-614.

Bronstein, O.; Kroh, A. The first mitochondrial genome of the model echinoid *Lytechinus variegatus* and insights into Odontophoran phylogenetics. *Genomics* **2018**, in press, doi: 10.1016/j.ygeno.2018.04.008

Cantatore, P.; Roberti, M.; Rainaldi, G.; Gadaleta, M.N.; Saccone, C. The complete nucleotide sequence, gene organization, and genetic code of the mitochondrial genome of *Paracentrotus lividus*. *J. Biol. Chem.* **1989**, *264*, 10965-10975.

Chen, Y.; Chang, Y.; Wang, X.; Qiu, X.; Liu, Y. De novo assembly and analysis of tissue-specific transcriptomes revealed the tissue-specific genes and profile of immunity from *Strongylocentrotus intermedius*. *Fish Shellfish Immunol*. **2015**, *46*, 723-736, doi: 10.1016/j.fsi.2015.07.013.

Cea, G.; Gaitan-Espitia, J.D.; Cardenas, L. Complete mitogenome of the edible sea urchin *Loxechinus albus*: genetic structure and comparative genomics within Echinozoa. *Mol. Biol. Rep*. 2015, *42*, 1081-1089.

De Giorgi, C.; Martiradonna, A.; Lanave, C.; Saccone, C. Complete sequence of the mitochondrial DNA in the sea urchin *Arbacia lixula*: conserved features of the echinoid mitochondrial genome. *Mol. Phylogenet. Evol*. **1996**, *5*, 323-332.

Dilly, G.F.; Gaitan-Espitia, J.D.; Hofmann, G.E. Characterization of the Antarctic sea urchin (*Sterechinus neumayeri*) transcriptome and mitogenome: a molecular resource for phylogenetics, ecophysiology and global change biology. *Mol. Ecol. Resour*. **2015**, *15*, 425-436.

Ding, J.; Yang, D.; Chang,Y.; Wang, Y.; Weijie Zhang, W.; Chen, T. Comparative transcriptome analysis of tube feet of different colors in the sea urchin *Strongylocentrotus intermedius*. *Genes Genom.* **2017**, *39*, 1215–1225.

Ding, J.; Liu, X.; Chang, Y.; Zhang, W. The complete mitochondrial genome sequence of the sea urchin. *Glyptocidaris crenularis* and its phylogenetic analysis. *Conserv. Genet. Resour*. **2017**, *9*, 63–66.

Fan, S.; Hu, C.; Wen, J.; Zhang, L. Characterization of mitochondrial genome of sea cucumber *Stichopus horrens*: a novel gene arrangement in Holothuroidea. *Sci. China Life Sci.* **2011**, *54*, 434-441.

Fu, W.; Liu, C.; Wu, G.; Zeng, X. The complete mitochondrial genome of *Temnopleurus hardwickii*. (Camarodonta: Temnopleuridae). *DNA Mapp. Seq. Anal.* **2014**, *27*, 3977-3978.

Gaitan-Espitia, J.D.; Hofmann, G.E. Mitochondrial genome architecture of the giant red sea urchin *Mesocentrotus franciscanus* (Strongylocentrotidae, Echinoida). *DNA Mapp. Seq. Anal.* **2016**, *27*, 591-592.

Huo,Y.-Y.; Xu, X.-W.; Wang, C.-S. Mitochondrial genome of the deep-sea sea cucumber *Peniagone* sp. from the Mariana Trench. Unpublished.

Jacobs, H.T.; Elliott, D.J.; Math, V.B.; Farquharson, A. Nucleotide sequence and gene organization of sea urchin mitochondrial DNA. *J. Mol. Biol*. **1988**, *202*, 185-217.

Jung, G.; Choi, H.J.; Pae, S.; Lee, Y.H. Complete mitochondrial genome of sea urchin: *Mesocentrotus nudus* (Strongylocentrotidae, Echinoida). *Mitochondr. DNA* **2013**, *24*, 466-468.

Jia, Z.; Wang, Q.; Wu, K.; Wei, Z.; Zhou, Z.; Liu, X. De novo transcriptome sequencing and comparative analysis to discover genes involved in ovarian maturity in *Strongylocentrotus nudus*. *Comp. Biochem. Physiol. D Genom. Proteom*. **2017**, *23*, 27-38, doi: 10.1016/j.cbd.2017.05.002.

Jung, G.; Lee, Y-H. Molecular phylogeny and mitochodrial DNA sequence evolution of *Strongylocentrotidae*, Echinoida. Unpublished.

Jung, G.; Choi, H.J.; Myoung, J.G.; Lee, Y.H.. Complete mitochondrial genome of sea urchin: *Hemicentrotus pulcherrimus* (Camarodonta, *Strongylocentrotidae*). *Mitochondr. DNA* **2014**, *25*, 439-440.

Jung, G.; Lee, Y-H. Complete mitochondrial genome of Chilean sea urchin: *Loxechinus albus* (Camarodonta, *Parechinidae*). *Mitochondr. DNA* **2015**, *26*, 883-884.

Jung, G.; Kim, C.G.; Lee, Y.H. Complete mitochondrial genome sequence of *Heliocidaris crassispina* (Camarodonta, Echinometridae). *DNA Mapp. Seq. Anal.* **2016**, *27*, 2393-2394.

Jung, G.; Park, H.Y.; Kim, K.S.; Lee, M-Y; Lee, H.; Lee, H.S.; Han, S-H.; Kim, C-B. Population genetic structure of spotted seal (*Phoca largha*) based on mitochondrial DNA sequences including Korean populations. Unpublished.

Ketchum, R.N.; DeBiasse, M.B.; Ryan, J.F.; Burt, J.A.; Reitzel, A.M. The complete mitochondrial genome of the sea urchin, *Echinometra* sp. EZ. *Mitochondrial DNA Part B: Res.* **2018**, *3*, 1225-1227.

Ketchum, R.N.; DeBiasse, M.B.; Ryan, J.F.; Burt, J.A.; Reitzel, A.M. Data from: The complete mitochondrial genome of the sea urchin, *Echinometra* sp. EZ. *Dryad Digital Repository* **2018**, https://doi.org/10.5061/dryad.20k3fj0.

Kober, K.M.; Bernardi, G. Phylogenomics of strongylocentrotid sea urchins. *BMC Evol. Biol*. **2013**, *13*, 88.

Láruson, A.J. Rates and relations of mitochondrial genome evolution across the Echinoidea, with special focus on the superfamily Odontophora. *Ecol. Evol*. **2017**, *7*, 4543-4551.

Li, C.; Wu, G.; Fu, W.; Zeng, X. The complete mitochondrial genome of *Diadema setosum* (Diadematacea: Diadematidae). *Mitochondrial DNA Part B: Res.* **2016**, *1*, 873-874.

Li, C.; Wu, G.; Fu, W.; Zeng, X. The complete mitochondrial genome of *Echinothrix diadema* (Diadematacea: *Diadematidae*). Unpublished.

Lin, J-P.; Tsai, M-H.; Kroh, A.; Trautman, A.; Machado, D.J.; Chang, L-Y.; Reid, R.; Lin, K-T.; Bronstein, O.; Lee, S-J.; Janies, D. The complete mitochondrial genome of *Sinaechinocyamus mai* (Echinoidea: Clypeasteroidea) and its implications for echinoid lantern evolution. *Gene* **2018**, in press.

Liu, W. Complete mitochondrial genome of the *Parastichopus californicus*. Unpublished.

Matsubara, M.; Komatsu, M.; Araki, T.; Asakawa, S.; Yokobori, S.; Watanabe, K.; Wada, H. The phylogenetic status of Paxillosida (Asteroidea) based on complete mitochondrial DNA sequences. *Mol. Phylogenet. Evol*. **2005**, *36*, 598-605.

Mu, W.; Liu, J.; Zhang, H. The first complete mitochondrial genome of the Mariana Trench Freyastera benthophila (Asteroidea: Brisingida: Brisingidae) allows insights into the deep-sea adaptive evolution of Brisingida. *Ecol. Evol*. **2018**, *8*, 10673–10686.

Murali, S.; Liu, Y.; Vee, V.; English, A.; Wang, M.; Skinner, E.; Han, Y.; Muzny, D. M.; Worley, K. C.; Gibbs, R. A. *Eucidaris tribuloides* genome sequencing project. Unpublished.

Nam, W.S.; Kanno, M.; Kijima, A. *Strongylocentrotus nudus* NADH:ubiquinone oxidoreductase 6 (ND6) gene, partial cds; mitochondrial gene for mitochondrial product. Unpublished.

Park, H.J.; Ryu, S.H.; Hwang, U.W. Complete mitochondrial genome of *Psedomaretia alta* (Echinodermata, Echinoidea, Spatangidae). Unpublished.

Perseke, M.; Fritzsch, G.; Ramsch, K.; Bernt, M.; Merkle, D.; Middendorf, M.; Bernhard, D.; Stadler, P.F.; Schlegel, M. Evolution of mitochondrial gene orders in echinoderms. *Mol. Phylogenet. Evol*. **2008**, *47*, 855-864.

Perseke, M.; Bernhard, D.; Fritzsch, G.; Brummer, F.; Stadler, P.F.; Schlegel, M. Mitochondrial genome evolution in Ophiuroidea, Echinoidea, and Holothuroidea: insights in phylogenetic relationships of Echinodermata. *Mol. Phylogenet. Evol*. **2010**, *56*, 201-211.

Rhee, J.S.; Ki, J.S.; Hwang, D.S.; Park, H.; Ahn, I.Y.; Lee, J.S. Complete mitochondrial genome of the Arctic green sea urchin *Strongylocentrotus droebachiensis* (Strongylocentrotidae, Echinoidea). *Mitochondr. DNA* **2012**, *23*, 369-370.

Sasaki, M.; Hamaguchi, M. *Parastichopus nigripunctatus* mitochondrion, complete genome. Unpublished

Scouras, A.; Smith, M.J. A novel mitochondrial gene order in the crinoid echinoderm *Florometra serratissima*. *Mol. Biol. Evol*. **2001**, *18*, 61-73.

Scouras, A.; Beckenbach, K.; Arndt, A.; Smith, M.J. Complete mitochondrial genome DNA sequence for two ophiuroids and a holothuroid: the utility of protein gene sequence and gene maps in the analyses of deep deuterostome phylogeny. *Mol. Phylogenet. Evol*. **2004**, *31*, 50-65.

Scouras, A.; Smith, M.J. The complete mitochondrial genomes of the sea lily *Gymnocrinus richeri* and the feather star *Phanogenia gracilis*: signature nucleotide bias and unique nad4L gene rearrangement within crinoids. *Mol. Phylogenet. Evol*. **2006**, *39*, 323-334.

Seixas,V.C.; Ventura, C.R.R.; Paiva, P.C. The complete mitochondrial genome of the sea star *Echinaster* (Othilia) *brasiliensis* (Asteroidea: Echinasteridae). *Conserv. Genet. Resour*. **2019**, *11*, 151–155.

Shen, X.; Tian, M.; Liu, Z.; Cheng, H.; Tan, J.; Meng, X.; Ren, J. Complete mitochondrial genome of the sea cucumber *Apostichopus japonicus* (Echinodermata: Holothuroidea): the first representative from the subclass Aspidochirotacea with the echinoderm ground pattern. *Gene* **2009**, *439*, 79-86.

Smith, M.J.; Arndt, A.; Beckenbach, K.; Scouras, A. Closing the Echinoderm Circle: the Complete mitochondria genome sequence of a sea cucumber and a brittle star. Submitted 18-OCT-2000. Unpublished.

Smith, M. *Pisaster ochraceus* mitochondrial DNA for NADH dehydrogenase subunits 1, 2, 3 and 4L, cytochrome oxidase subunits I, II and III, ATPase subunits 6 and 8, and 13 tRNAs. Direct submission (06-JAN-2003).

Sun, Z.H.; Zhang, J.; Zhang, W.J.; Chang, Y.Q. Gonadal transcriptomic analysis and identification of candidate sex-related genes in *Mesocentrotus nudus*. *Gene* **2019**, *698*, 72-81, doi: 10.1016/j.gene.2019.02.054.

Takano, T.; Ijichi, M.; Itoh, H.; Fukuda, H.; Yoshizawa, S. Complete mitochondrial genome sequences of a deep-sea holothurian species of the genus Scotoplanes (Elasipodida: Elpidiidae) *Mitochondrial DNA Part B: Res.* **2018**, *4*, 112-113.

Tang, M.; Tan, M.; Meng, G.; Yang, S.; Su, X.; Liu, S.; Song, W.; Li, Y.; Wu, Q.; Zhang, A.; Zhou, X. Multiplex sequencing of pooled mitochondrial genomes-a crucial step toward biodiversity analysis using mito-metagenomics. *Nucleic Acids Res*. **2014**, *42*, e166, doi: 10.1093/nar/gku917.

Wu, G.; Fu, W.; Zeng, X. The complete mitochondrial genome of *Salmacis sphaeroides*. Unpublished.

Wu, G.; Fu, W.; Zeng, X. The complete mitochondrial genome of *Temnopleurus reevesii*. Unpublished.

Wu, G.; Fu, W.; Zeng, X. The complete mitochondrial genome of *Salmacis bicolor*. Unpublished.

Wu, G.; Fu, W.; Zeng, X. The complete mitochondrial genome of *Temnopleurus toreumaticus* (Camarodonta: Temnopleuridae). Unpublished.

Xia, J.; Ren, C.; Yu, Z.;Wu, X.; Qian, J.; Hu, C. Complete mitochondrial genome of the sandfish Holothuria scabra. (Holothuroidea, Holothuriidae). *DNA Mapp. Seq. Anal.* **2015**, *27*, 4174-4175.

Yasuda, N.; Hamaguchi, M.; Sasaki, M.; Nagai, S.; Saba, M.; Nadaoka, K. Complete mitochondrial genome sequences for Crown-of-thorns starfish *Acanthaster planci* and *Acanthaster brevispinus*. *BMC Genomics* **2006**, *7*, 17, doi: 10.1186/1471-2164-7-17.

Zhan, Y.; Li, J.; Sun, J.; Zhang, W.; Li, Y.; Cui, D.; Hu, W.; Chang, Y. The Impact of Chronic Heat Stress on the Growth, Survival, Feeding, and Differential Gene Expression in the Sea Urchin *Strongylocentrotus intermedius*. *Front. Genet*. **2019**, *10*, 301, doi: 10.3389/fgene.2019.00301.

Zhang, X.; Wang, C.; Li, X*. Parastichopus parvimensis* mitochondrion, complete genome. Direct Submission (18-NOV-2015).

Zhang, W.; Lv Z.; Li C.; Sun Y.; Jiang H.; Zhao M.; Zhao X.; Shao Y.; Chang Y. Transcriptome profiling reveals key roles of phagosome and NOD-like receptor pathway in spotting diseased Strongylocentrotus intermedius. *Fish Shellfish Immunol*. **2019**, *84*, 521-531, doi: 10.1016/j.fsi.2018.10.042.

Zhao, C.; Ding, J.; Yang, M.; Shi, D.; Yin, D.; Hu, F.; Sun, J.; Chi, X.; Zhang, L.; Chang, Y. Transcriptomes reveal genes involved in covering and sheltering behaviors of the sea urchin Strongylocentrotus intermedius exposed to UV-B radiation. *Ecotoxicol. Environ. Saf*. **2019**, *167*, 236-241, doi: 10.1016/j.ecoenv.2018.10.031.
