# Supplementary material for: Trans-Species Polymorphism in Mitochondrial Genome of Camarodont Sea Urchins
Source: Genes (Basel). 2019 Aug 5;10(8):592. doi: 10.3390/genes10080592 (PMC6723515; doi:10.3390/genes10080592)
Supplement: Supplementary file 1 [file genes-10-00592-s001.zip › Table_S2.doc]

**Table S2.** The ND6 C-terminal sequences of sea urchins studied at present work

| Species | ND6 C-terminal end | | | | | | | | | | | | | | | | | | | | | N |
| --- | --- | --- | --- | --- | --- | --- | --- | --- | --- | --- | --- | --- | --- | --- | --- | --- | --- | --- | --- | --- | --- | --- |
| *Strongylocentrotus pallidus* (AM900392.1, Echinidea) | **R** | | | **A** | | | **I** | | | **W** | | | **L** | | | **W** | | | **Stop** | | | 165 |
|  | C | G | T | G | C | T | A | T | T | **T** | **G** | **G** | T | T | A | T | G | A | T | A | A |  |
| *Strongylocentrotus droebachiensis* (AM900391.1, Echinidea) | **R** | | | **A** | | | **I** | | | **W** | | | **L** | | | **W** | | | **Stop** | | | 165 |
|  | C | G | C | G | C | T | A | T | T | **T** | **G** | **G** | T | T | A | T | G | A | T | A | A |  |
| *Strongylocentrotus fragilis* (KC8982002.2, Echinidea) | **R** | | | **A** | | | **I** | | | **W** | | | **L** | | | **W** | | | **Stop** | | | 165 |
|  | C | G | C | G | C | T | A | T | C | **T** | **G** | **G** | T | T | A | T | G | A | T | A | A |  |
| *Strongylocentrotus purpuratus* (X12631.1, Echinidea) | **R** | | | **A** | | | **I** | | | **W** | | | **L** | | | **W** | | | **Stop** | | | 165 |
|  | C | G | T | G | C | T | A | T | C | **T** | **G** | **G** | T | T | A | T | G | A | T | A | A |  |
| *Strongylocentrotus intermedius* (KY964299.1, Echinidea) | **R** | | | **A** | | | **I** | | | **W** | | | **L** | | | **W** | | | **Stop** | | | 165 |
|  | C | G | C | G | C | T | A | T | T | **T** | **G** | **G** | T | T | A | T | G | A | T | A | A |  |
| *Strongylocentrotus intermedius* (KY964300.1, Echinidea) | **R** | | | **A** | | | **I** | | | **Stop** | | | **−** | | | **−** | | | **−** | | | 162 |
|  | C | G | C | G | C | T | A | T | T | **T** | **A** | **G** | **−** | **−** | **−** | **−** | **−** | **−** | **−** | **−** | **−** |  |
| *Hemicentrotus pulcherrimus* (KC490911.1, Echinidea) | **R** | | | **A** | | | **I** | | | **Stop** | | | **−** | | | **−** | | | **−** | | | 162 |
|  | C | G | T | G | C | T | A | T | T | **T** | **A** | **G** | **−** | **−** | **−** | **−** | **−** | **−** | **−** | **−** | **−** |  |
| *Pseudocentrotus depressus* (KC490913.1, Echinidea) | **R** | | | **A** | | | **I** | | | **W** | | | **L** | | | **W** | | | **Stop** | | | 165 |
|  | C | G | T | G | C | T | A | T | T | **T** | **G** | **G** | T | T | A | T | G | A | T | A | A |  |
| *Mesocentrotus nudus* (JX263663.1, Echinidea) | **R** | | | **A** | | | **I** | | | **W** | | | **L** | | | **W** | | | **Stop** | | | 165 |
|  | C | G | T | G | C | T | A | T | T | **T** | **G** | **G** | T | T | A | T | G | A | T | A | A |  |
| *Mesocentrotus nudus* (AB863099.1, Echinidea) | **R** | | | **A** | | | **I** | | | **Stop** | | | **−** | | | **−** | | | **−** | | | 162 |
|  | C | G | T | G | C | T | A | T | T | **T** | **A** | **G** | **−** | **−** | **−** | **−** | **−** | **−** | **−** | **−** | **−** |  |
| *Mesocentrotus franciscanus* (KJ526170.1, Echinidea) | **R** | | | **A** | | | **I** | | | **Stop** | | | **−** | | | **−** | | | **−** | | | 162 |
|  | C | G | T | G | C | T | A | T | T | **T** | **A** | **G** | **−** | **−** | **−** | **−** | **−** | **−** | **−** | **−** | **−** |  |
| *Heliocidaris crassispina* (KC479025.1, Echinidea) | **R** | | | **A** | | | **I** | | | **W** | | | **L** | | | **W** | | | **Stop** | | | 165 |
|  | C | G | T | G | C | T | A | T | T | **T** | **G** | **G** | T | T | A | T | G | A | T | A | G |  |
| *Heterocentrotus mamillatus* (KJ680292.1, Echinidea) | **R** | | | **A** | | | **I** | | | **L** | | | **L** | | | **W** | | | **Stop** | | | 165 |
|  | C | G | T | G | C | A | A | T | T | **T** | **T** | **G** | T | T | A | T | G | A | T | A | A |  |
| *Echinometra mathaei* (KJ680291.1, Echinidea) | **R** | | | **A** | | | **I** | | | **Stop** | | | **−** | | | **−** | | | **−** | | | 162 |
|  | C | G | T | G | C | A | A | T | T | **T** | **A** | **G** | **−** | **−** | **−** | **−** | **−** | **−** | **−** | **−** | **−** |  |
| *Tripneustes gratilla* (KJ680294.1, Echinidea) | **R** | | | **A** | | | **I** | | | **Stop** | | | **−** | | | **−** | | | **−** | | | 162 |
|  | C | G | T | G | C | T | A | T | T | **T** | **A** | **G** | **−** | **−** | **−** | **−** | **−** | **−** | **−** | **−** | **−** |  |
| *Lytechinus variegatus* (MG676468.1, Echinidea) | **R** | | | **A** | | | **I** | | | **Stop** | | | **−** | | | **−** | | | **−** | | | 162 |
|  | C | G | G | G | C | T | A | T | T | **T** | **A** | **G** | **−** | **−** | **−** | **−** | **−** | **−** | **−** | **−** | **−** |  |
| *Paracentrotus lividus* (J04815.1, Echinidea) | **R** | | | **A** | | | **I** | | | **Stop** | | | **−** | | | **−** | | | **−** | | | 160 |
|  | C | G | T | G | C | A | A | T | T | **T** | **A** | **G** | **−** | **−** | **−** | **−** | **−** | **−** | **−** | **−** | **−** |  |
| *Sterechinus neumayeri* (KF214257.1, Echinidea) | **R** | | | **A** | | | **I** | | | **Stop** | | | **−** | | | **−** | | | **−** | | | 162 |
|  | C | G | T | G | C | A | A | T | T | **T** | **A** | **G** | **−** | **−** | **−** | **−** | **−** | **−** | **−** | **−** | **−** |  |
| *Loxechinus albus* (KC490910.1, Echinidea) | **R** | | | **A** | | | **I** | | | **Stop** | | | **−** | | | **−** | | | **−** | | | 162 |
|  | C | G | T | G | C | A | A | T | T | **T** | **A** | **G** | **−** | **−** | **−** | **−** | **−** | **−** | **−** | **−** | **−** |  |
| *Temnopleurus reevesii* (KU302106.1, Temnopleuridea) | **R** | | | **A** | | | **I** | | | **Stop** | | | **−** | | | **−** | | | **−** | | | 162 |
|  | C | G | T | G | C | T | A | T | T | **T** | **A** | **G** | **−** | **−** | **−** | **−** | **−** | **−** | **−** | **−** | **−** |  |
| *Mespilia globulus* (KJ680293.1, Temnopleuridea) | **R** | | | **A** | | | **I** | | | **L** | | | **L** | | | **C** | | | **Stop** | | | 165 |
|  | C | G | T | G | C | T | A | T | T | **T** | **T** | **G** | T | T | A | T | G | T | T | A | G |  |
| *Salmacis sphaeroides* (KU302103.1, Temnopleuridea) | **R** | | | **A** | | | **I** | | | **Stop** | | | **−** | | | **−** | | | **−** | | | 162 |
|  | C | G | T | G | C | C | A | T | T | **T** | **A** | **G** | **−** | **−** | **−** | **−** | **−** | **−** | **−** | **−** | **−** |  |
| *Salmacis bicolor* (KU302104.1, Temnopleuridea) | **R** | | | **A** | | | **I** | | | **Stop** | | | **−** | | | **−** | | | **−** | | | 162 |
|  | C | G | T | G | C | T | A | T | T | **T** | **A** | **G** | **−** | **−** | **−** | **−** | **−** | **−** | **−** | **−** | **−** |  |
| *Temnopleurus toreumaticus* (KU302105.1, Temnopleuridea) | **R** | | | **A** | | | **I** | | | **Stop** | | | **−** | | | **−** | | | **−** | | | 162 |
|  | C | G | T | G | C | T | A | T | T | **T** | **A** | **G** | **−** | **−** | **−** | **−** | **−** | **−** | **−** | **−** | **−** |  |
| *Temnopleurus hardwickii* (KP070768.1, Temnopleuridea) | **R** | | | **A** | | | **I** | | | **Stop** | | | **−** | | | **−** | | | **−** | | | 162 |
|  | C | G | T | G | C | T | A | T | T | **T** | **A** | **G** | **−** | **−** | **−** | **−** | **−** | **−** | **−** | **−** | **−** |  |
| *Glyptocidaris crenularis* (KX638403.1, Stomopneustoida) | **R** | | | **A** | | | **I** | | | **Stop** | | | **−** | | | **−** | | | **−** | | | 162 |
|  | C | G | T | G | C | T | A | T | T | **T** | **A** | **G** | **−** | **−** | **−** | **−** | **−** | **−** | **−** | **−** | **−** |  |
| *Arbacia lixula* (X80396.1, Arbacioida) | **Q** | | | **A** | | | **I** | | | **Stop** | | | **−** | | | **−** | | | **−** | | | 162 |
|  | C | A | G | G | C | T | A | T | C | **T** | **A** | **A** | **−** | **−** | **−** | **−** | **−** | **−** | **−** | **−** | **−** |  |
| *Nacospatangus altus* (KC990834.1, Spatangoida) | **R** | | | **A** | | | **I** | | | **Stop** | | | **−** | | | **−** | | | **−** | | | 162 |
|  | C | G | A | G | C | T | A | T | T | **T** | **A** | **G** | **−** | **−** | **−** | **−** | **−** | **−** | **−** | **−** | **−** |  |
| *Echinocardium cordatum* (FN562581.1, Spatangoida) | **R** | | | **A** | | | **I** | | | **Stop** | | | **−** | | | **−** | | | **−** | | | 162 |
|  | C | G | T | G | C | T | A | T | T | **T** | **A** | **G** | **−** | **−** | **−** | **−** | **−** | **−** | **−** | **−** | **−** |  |
| *Diadema setosum* (KX385835.1, Diadematoida) | **R** | | | **A** | | | **L** | | | **Stop** | | | **−** | | | **−** | | | **−** | | | 162 |
|  | C | G | G | G | C | A | C | T | T | **T** | **A** | **G** | **−** | **−** | **−** | **−** | **−** | **−** | **−** | **−** | **−** |  |
| *Echinothrix diadema* (KX385836.1, Diadematoida) | **R** | | | **A** | | | **L** | | | **Stop** | | | **−** | | | **−** | | | **−** | | | 162 |
|  | C | G | G | G | C | G | C | T | T | **T** | **A** | **G** | **−** | **−** | **−** | **−** | **−** | **−** | **−** | **−** | **−** |  |
| **Cucumaria miniata* (AY182376, Holothuroidea, Cucumariidae) | **R** | | | **A** | | | **L** | | | **Stop** | | | **−** | | | **−** | | | **−** | | | 162 |
|  | C | G | T | G | C | T | T | T | A | **T** | **A** | **A** | **−** | **−** | **−** | **−** | **−** | **−** | **−** | **−** | **−** |  |

Conceptual translation of the ND6 gene shown in one-letter abbreviations (echinoderm mitochondrial genetic code). Last three ND6 C-terminal amino acids of the S variant and last six ND6 C-terminal amino acids of the L variant are included. Amino acids are in bold face. The codons including the 488A>G polymorphic site and stop codons are in red bold. The sea urchin species are listed in accordance with the maximum likelihood tree based on the complete mitochondrial genomes (Fig. 2). The GenBank accession numbers and the taxonomical infraorders (Echinidea and Temnopleuridea) and orders (Stomopneustoida, Arbacioida, Spatangoida, and Diadematoida) are in parentheses (Kroh and Smith 2010). There are two length variants of ND6, including 162 (S variant) and 165 (L variant) amino acids. The ND6 sequence of *Mesocentrotus nudus* with the GenBank accession number AB863103 is longer than other sequences belonging to the L variant due to two additional amino acids (ND). The ND6 sequence of *Paracentrotus lividus* (J04815) is two amino acids shorter than the S variant due to internal deletions (positions # 125 and 131 in an alignment, Fig. S1). *Cucumaria miniata* (AY182376, Holothuroidea, Cucumariidae) is used as an outgroup (indicated with a star symbol). N: number of amino acids. *Eucidaris tribuloides* (JZLH01S0471648.1, order Cidaroida) is not included to the table due to deletion of seven amino acids at the ND6 C-terminal end (see Fig. S1). Stop (in bold face) means a termination stop codon.

**References**

Kroh A, Smith AB. 2010. The phylogeny and classification of post-Palaeozoic echinoids. Journal of Systematic Palaeontology. Vol. 8, Issue 2. P. 147-212.
