# Supplementary material for: Trans-Species Polymorphism in Mitochondrial Genome of Camarodont Sea Urchins
Source: Genes (Basel). 2019 Aug 5;10(8):592. doi: 10.3390/genes10080592 (PMC6723515; doi:10.3390/genes10080592)
Supplement: Supplementary file 1 [file genes-10-00592-s001.zip › Figure_S1.doc]

Figure S1. The ND6 protein alignment of sea urchins

```
-----------------------------------------------------------------------------------------------------------------------------------------------------------------------------------
                                                                                                                 111111111111111111111111111111111111111111111111111111111111111111
                       111111111122222222223333333333444444444455555555556666666666777777777788888888889999999999000000000011111111112222222222333333333344444444445555555555666666
              123456789012345678901234567890123456789012345678901234567890123456789012345678901234567890123456789012345678901234567890123456789012345678901234567890123456789012345
SP_AM900392   MVVYLTLIVMLFGSTLVFYSLSPYYSALGLVVVSVSGCLILSFLGSSFVAIVLLLVYMGGMLVVFAYSSAISAERFPSVNNLGEVVGLSFLFSSWVFMSFDNFQDLSNTFYCFVSGESLVGSSTFYNSGGVLVILGVFVLLIALVGALIISRGIESTIIRAIWLW
SD_AM900391   MVVYLTLIVMLFGSTLVFYSLSPYYSALGLVVVSVSGCLVLSFLGSSFVAIVLLLVYMGGMLVVFAYSSAISAERFPSVNNLGEVVGLSFLFSSWVFMSFDNFQDLSNTFHCFISGESLVGSSTFYNSGGVLVILGVFVLLIALVGALIISRGIESTIIRAIWLW
SF_KC898200   MVVYLTLIVMLFGSTLVFYSLSPYYSALGLVVVSVSGCLVLSFLGSSFVAIVLLLVYMGGMLVVFAYSSAISAERFPSVNNLGEVVGLSFLFSSWVFMSFDNFQDLSNTFHCFISGESLVGSSTFYNSGGVLVILGVFVLLIALVGALIISRGIESTIIRAIWLW
SI_KY964299   MVVYLTLIVMLFGSTLVFYSLSPYYSALGLVVVSVSGCLVLSFLGSSFVAIVLLLVYMGGMLVVFAYSSAISAERFPSVNNLGEVVGLSFLFSSWVFISFDNFQDLSNTFHCFIGGESLVGSSTFYNSGGVLVVLGVFVLLIALVGALVISRGIESTIIRAIWLW
SI_KY964300   MVVYLTLIVMLFGSTLVFYSLSPYYSALGLVVVSVSGCLVLSFLGSSFVAIVLLLVYMGGMLVVFAYSSAISAERFPSVNNLGEVVGLSFLFSSWVFISFDNFQDLSNTFHCFIGGESLVGSSTFYNSGGVLVVLGVFVLLIALVGALVISRGIESTIIRAI---
SP_X12631     MVVYVTLIVMLFGSTLVFYSLSPYYSALGLVVFSVPGSFVLSFLGSSFVPIVLFLVYIGGMLVVFPYSSAISPERFPSVNNLGEVVGLVFLFSSWVFMSFDNFQDLKNIFHCFVSGESLVGSNTFYNSGGVLVILGVFVLLVALVGALIISRGIESTIIRAIWLW
HP_KC490911   MVIYLTLMVMLFGSTLVFYSLSPYYSALGLVVVSVSGCLVLSFLGSSFVAIVLLLVYMGGMLVVFAYSSAISAERFPSINNLGEVVGLSFLFSSWVFISFDNFQDISNTFYCFISGESLVGSSTLYSSGGVLVILGVFVLLVALVGALIISRGIESTIIRAI---
MN_JX263663   MVVYLTLIVMLFGSTLVFYSLSPYYSALGLVIVSVSGCLVLSFLGSSFIAIVLLLVYMGGMLVVFAYSSAISAERFPSVNNLGEVVGLSVLFSSWVFMSFDNFQDLSNTFHCFVSGESLIGSSTFYSSGGVLVILGVFVLLIALVGALIISRGIESTIIRAIWLW
MN_AB863099   MVVYLTLIVMLFGSTLVFYSLSPYYSALGLVIVSVSGCLVLSFLGSSFIAIVLLLVYMGGMLVVFAYSSAISAERFPSVNNLGEVVGLSVLFSSWVFMSFDNFQDLSNTFHCFVSGESLIGSSTFYSSGGVLVILGVFVLLIALVGALIISRGIESTIIRAI---
PD_KC490913   MVVYLTLIVMLFGSTLVFYSLSPYYSALGLVIVSVSGCLVLSFLGSSFVAIVLLLVYMGGMLVVFAYSSAISAERFPSVNNLGEVIGLSVLFSSWVFMSFDNFQDFSNAFYCFVSGESLVGSSTLYSSGGVLVVLGVFVLLIALVGALIISRGIESTIIRAIWLW
MF_MG676467   MVVYLTLIVMLFGSTLVFYSLSPYYSALGLVIVSVSGCLVLSFLGSSFVAIVLLLVYMGGMLVVFAYSSAISAERFPSVNNLGEVVGLSVLFSSWVFMSFDNFQDFGNTFYCFVSGESLIGSSTFYSSGGVLVVLGVFVLLIALVGALIISRGIESTIIRAI---
HC_KC479025   MVVYLTLVMMLFGSTLVFYSLSPYYSALGLVIVSVSGCCVLSLLGGSFVALVLLLVYMGGMLVVFAYSSAISAERFPSVNNLSEVIGLSVFLSSWVFMSFDNFQDFGNVFYCLVSGESLLGSSTFYSSGGVLVVLGVFVLLIALVGALIISRGIESTVIRAIWLW
HM_KJ680292   MTVYLTLVMMLFGSTLVFYSLSPYYSALGLVIVSVSGCSVLSLLGGSFIALVLLLVYMGGMLVVFAYSSAISAERFPSVNNLSEVIGLSILLSSWVFLSFDNFQDFNNFFNCIVSGESLVGSSTFYSSGGVLVILGVFVLLIALVGALIISRGIESTVIRAILLW
EM_KJ680291   MVVYLMFVMMLFGSTLVFYSLSPYYSALGLVVVSVSGCSVLSLLGGSFIALVLLLVYMGGMLVVFAYSSAISAERFPSINNLSEVVGLSVFLSSWVFLSFDNFHDLSNTFGCIVSGESLVGSSTFYNDGGLLVIVGVFVLLIALVGALIISRGVESTVIRAI---
LV_MG676468   MVVYLSLVIMLFGSTLVFYSLSPYYSALGLVIVSVSGCLVLSFLGGSFVALVLLLVYMGGMLVVFAYSSAISAERFPSVNNLGEVIGLSFLLSSWVFMSFDNFQDCGNVFYCLVSGESLVGSGTLYGSGGVLAILGVFVLLIALVGALIISRGIESTVIRAI---
TG_KY268294   MVVYLSLVMMLFGSTLVFYSLSPYYSALGLVIVSVSGCLVLSLLGGSFVALVLLLVYMGGMLVVFAYSSAISAERFPSVNNLGEVVGLSVLLSSWVFVSFDNFQDGGNAFYCLVSGESLVGSGTLYGSGGVLAILGVFVLLIALIGALIISRGIESTVIRAI---
SN_KJ680295   MVVYLTLVMMLFGSTLVFYSLSPYYSALGLVIVSVSGCSILSLLGGSFIALVLLLVYMGGMLVVFAYSSAISADRFPSVNNFGEVIGLSVLFSSWIFFSFDNFQDFSNIFHSFINGESLIGSSTFYSEGGVLVLVGVFVLLVALVGALIISRGIESTVIRAI---
LA_KC490910   MALYLTLVMMLFGSTLVFYSLSPYYSAIGLVIVSVSGCVVLSFLGGSFVALVLLLVYMGGMLVVFAYSSAISAERFPSVNNLGEVIGLSVLFASWIFLSFDNFQDFSNVFFCFINGESLIGSSTFYSSGGVLVLVGVFVLLIALIGALIISRGIESTVIRAI---
PL_J04815     MALYLILVLMLFGSTLVFYSLSPYYSALGLVVVSISGCIILSFLGGSFVALVLVLVYMGGMLVVFAYSSAISAERFPSVNNLGEVIGLSVLFSSWVFLSFDNFQDFSNSFSCFISGESLVEAG-FY-MKGFLVLVGVFVLLVVLVGALIISRGIENTVIRAI---
SS_KU302103   MIIYLTLVMMLFGSTLVFYSFSPYFSALGLVVVSVSGCVSLSLMGGPFISLVLLLVYMGGMLVVFAYSSAISAERFPVVNNLGEVAGLSVLLSSWVFLSFDNFQEFGNFFYSFVSGESLLGSSTFYNSGGVLVIIGVFVLLVALVGALIISRGVENSIIRAI---
SB_KU302104   MVVYLTFVMMLFGSTLVFYSFSPYFSALGLVIVSVSGCISLSLMGSPFISLILLLVYMGGMLVVFAYSSAISAERFPVVNNLGEVAGLSFLLSSWAFVSFDNFQEFSNYFYSFVSGESLLGSSTFYNSGGVLVVLGVFVLLVALVGALIISRGVENSIIRAI---
TR_KU302106   MVIYLTLVMMLFGSTLVFYSFSPYFSALGLVIVSVSGCVILSLMGGSFISLVLLLVYMGGMLVVFAYSSAISAERFPTVNNLGEVGGLSILLSSWVFLSFDNFQNLGNVYFCLISGESLLGSSTFYNSGGVLVILGVFVLLVALVGALIISRGVENTIIRAI---
MG_KJ680293   MILYLVLVMMLFGSTLVFYSFSPYFSALGLVVVSVSGCIILSLLGSSFVSLVLLLVYMGGMLVVFAYSSAISAERFPVVNNLGEVGGLSFLFSSWVFLSFDNFHSLDNFFYGLISGSSLVGSSTFYNEGGVLVVLGVFVLFVALIGALIISRGVENNIIRAILLC
TT_KU302105   MIIYFTFVMMLFGSTLVFYSFSPYFSALGLVVVSVSGCVSLSLMGSSFISLVLLLVYMGGMLVVFAYSSAISAERFPVVNNLGEVGGLSVLLSSWVFLSFDNFQDFSNYFFCFVSGESLLGSSTFYNGGGVLVILGVFVLLVALIGALVISRGVENNIIRAI---
TH_KP070768   MVIYFTLVMMLFGSTLVFYSFSPYFSALGLVIVSVSGCVSLSFIGSPFISLVLLLVYMGGMLVVFAYSSAISAERFPVVNNLGEVAGLSILLSSWVFLSFDNFQDFGNCFYSLVSGESLLGSSTFYNDGGVLVVLGVFVLLIALIGALVISRGVENNIIRAI---
GC_KX638403   MMVYLSLVFMLVGSTLVFYSLSPYYSALGLVVVSVSGCVVLSLLGGSFIALVLLLVYMGGMLVVFAYSSAISAERFPSVSNLGEVLGLSLFFSSWVFLSFDNFEELNNSFHCFISGESLIGSGTFYNEGGVLVVLGVFILLVALVGALIISRGIENTIIRAI---
AL_X80396     MIIYLSLVCLLVGSTLVFYSLSPYYSALGLVVVSISGCISLSLIGGSFIALVLLLVYMGGMLVVFAYSSAISAERFPSVSNISEVIGLSAFFSSWVFLSFDNLNETSNCSSTIISNEGVLGSSIFYNDGSVLVVLGVFILFIVLVGALIISRGAENNIVQAI---
NA_KC990834   MIIYLSLVLMLVGSTLVFYSLSPYYSALGLVVVSVSGCITLGALGGSFVALVLLLVYMGGMLVVFAYSSAISAERFPSVSNLGEIIGLSTFFSTWVFFSFDEFNETNNSFFQFVSGESILGSSTFYNDGGALVIIGVFILLVALVGALIISRGVESTIVRAI---
EC_FN562581   MVIYLCLVFMLVGSTLVFYSLSPYYSALGLVVVSVSGCVVLGVLGGSFVALVLLLVYMGGMLVVFAYSSAISAERFPSVSNLSEVVGLSLFFSTWVFVSFNDLNDTNNNFFQLISGESVLGSSSFYNEGGALVIVGVFILLVALVGALIISRGVESTIVRAI---
DS_KX385835   MVMYFSLVLMLIGSVLVFYSLSPYYSALGLVLVSVSGCVVLSLLGASFLALTLLIVYMGGMLVVFAYSSAISAERFPQVSNLSEVVSLSFLLCSWVFISFDNLEDGHFPHGGMCSGEVLLGSSSFYDFGGALVVIGVFVLLVALVGALIISRGIESSIIRAL---
ED_KX385836   MVMYFSLVFMLIGSVLVFYSLSPYYSALGLVLVSVSGCVVLSLLGTSFLALTLLIVYMGGMLVVFAYSSAISAERFPQVSNLSEVIGLSFLLCSWVFVSFDNLEDGYLPLQGMCSGEVLLGSSSFYDFGGALVIVGVFVLLIALVGALIISRGIESNIIRAL---
ET_JZLH01S-   MIVYFFLVLMLAGSTLVFYSLSPYYSALGLVIVAVSGCVVLGILGGAFVALILLLIYMGGMLVVFAYSSALSAERFPMVSNLSEILGLSLFFSSWVFVSFDNFQDFFFSAGNIVNNVGLGGSSTFYGVGGVLVLLGVYFVGCVSCCFNNFSRGSG----------
0471648.1
-----------------------------------------------------------------------------------------------------------------------------------------------------------------------------------
```

Species designation, SP: *Strongylocentrotus pallidus*, SD: *Strongylocentrotus droebachiensis*, SF: *Strongylocentrotus fragilis*, SP: *Strongylocentrotus purpuratus*, SI: *Strongylocentrotus intermedius*, HP: *Hemicentrotus pulcherrimus*, PD: *Pseudocentrotus depressus*, MN: *Mesocentrotus nudus*, MF: *Mesocentrotus franciscanus*, HC: *Heliocidaris crassispina*, HM: *Heterocentrotus mamillatus*, EM: *Echinometra mathaei,* LV: *Lytechinus variegates*, TG: *Tripneustes gratilla*, PL: *Paracentrotus lividus*, SN: *Sterechinus neumayeri*, LA: *Loxechinus albus*, TR: *Temnopleurus reevesii*, MG: *Mespilia globulus*, SS: *Salmacis sphaeroides*, SB: *Salmacis bicolor*, TT: *Temnopleurus toreumaticus*, TH: *Temnopleurus hardwickii*, GC: *Glyptocidaris crenularis*, AL: *Arbacia lixula*, NA: *Nacospatangus altus*, EC: *Echinocardium cordatum*, DS: *Diadema setosum*, ED: *Echinothrix diadema*, ET: *Eucidaris tribuloides*. The GenBank accession number follows the species designation. The sea urchin species are listed in accordance with the maximum likelihood tree based on the complete mitochondrial genomes (see Fig. 2). The both, L and S ND6 variants are presented for the *Strongylocentrotus intermedius* (SI_KY964299, SI_KY964300) and *Mesocentrotus nudus* (MN_JX263663, MN_AB863099).
